# Perceptions, attitudes, and barriers toward research among medical students in the United Arab Emirates: a national cross-sectional study

**DOI:** 10.3389/fmed.2025.1729448

**Published:** 2025-12-15

**Authors:** Ruthwik Duvuru, Syed Ali Bokhari, Mohamed A. Elshafey, Ahmed Hafez Mousa, Laila Zarnegar, Ibrahim Hassan Ibrar, Ans Ahmed Mahmood, Uvashree Shrinivas, Aena Khan, Vikas Bansal, Stefan S. Du Plessis, Faisal A. Nawaz

**Affiliations:** 1 College of Medicine, Mohammed Bin Rashid University of Medicine and Health Sciences, Dubai Health, Dubai, United Arab Emirates; 2 Al Amal Psychiatric Hospital, Emirates Health Services, Dubai, United Arab Emirates; 3 College of Medicine, University of Sharjah, Sharjah, United Arab Emirates; 4 Neurosciences Department, Dubai Health, Dubai, United Arab Emirates; 5 Department of Neurosurgery, Graduate Medical Education, Mohammed Bin Rashid University of Medicine and Health Sciences, Dubai Health, Dubai, United Arab Emirates; 6 Gulf Medical University, Ajman, United Arab Emirates; 7 RAK Medical and Health Sciences University, Ras Al Khaimah, United Arab Emirates; 8 College of Medicine, Dubai Medical University, Dubai, United Arab Emirates; 9 WellSpan Health, York, PA, United States

**Keywords:** medical education, student research, research barriers, mentorship, undergraduate, attitudes, perceptions, United Arab Emirates

## Abstract

**Background:**

Medical research is increasingly recognized as crucial for career progression, prompting institutions to embed research into curricula. Despite these efforts, barriers continue to impede medical students’ engagement in meaningful research. This comprehensive nationwide study explores medical students’ research perceptions, attitudes, and challenges across UAE medical universities, capturing insights from a significant and diverse student population.

**Methods:**

This cross-sectional study utilized an online self-administered questionnaire distributed to medical students enrolled at seven medical colleges across the UAE. Data collection was conducted over 6 months from May to October 2024. The questionnaire comprised 53 items rated on a 5-point Likert scale and was organized into four key domains: demographics, prior research experience, attitudes toward research, and perceived barriers. Data were analyzed using descriptive statistics to summarize participant characteristics and inferential analyses, including multivariate linear regression, to explore associations and predictors of research attitudes, experiences, and barriers. A *p*-value of < 0.05 was considered statistically significant.

**Results:**

A total of 612 medical students participated (68.8% male; 66.5% pre-clinical). While 68.3% reported prior research experience, only 15.7% had published. Most students agreed research should be integrated into medical curricula (91.0%) and is crucial for careers (88.4%), yet 19.8% found existing curricula insufficient. Primary barriers included lack of funding (46.4%), insufficient mentorship (35.6%), statistical skills deficits (47.7%), and limited protected research time (39.5%). Younger students (≤ 21 years) were curriculum vitae (CV)-driven and cited mentorship gaps (*p* = 0.009), while older students (≥ 24 years) reported funding constraints (*p* < 0.001) but achieved higher publication rates (*p* = 0.001). Males more frequently reported financial barriers (*p* = 0.007); females cited limited mentorship (*p* = 0.044). Senior students published more (*p* < 0.001) but struggled with balancing research with academics (*p* < 0.001). Multivariate regression identified age, research barriers, nationality, medical college, cumulative grade point average (CGPA), and prior publication as significant predictors of research attitudes and participation (all *p* < 0.05).

**Conclusion:**

Medical students in the UAE demonstrate positive attitudes toward research but face significant structural barriers, including inadequate funding, limited mentorship opportunities, and insufficient institutional support. Early structured research training, formal mentorship programs, and protected research time are essential interventions. Institutions should embed these elements longitudinally to cultivate a robust research culture and enhance undergraduate research outputs.

## 1 Introduction

Undergraduate research develops core clinical reasoning and appraisal skills, yet student engagement varies across settings. Research in medical education plays a pivotal role in shaping healthcare by enhancing students’ academic, clinical, and scientific abilities (1). The integration of research into medical curricula has been recognized as essential for developing evidence-based practitioners who contribute to medical science advancement (2). Undergraduate research provides students with critical thinking, analytical, and problem-solving skills essential for clinical decision-making and innovation (3). However, despite its benefits, the engagement of medical students in research remains variable across different regions and institutions (4).

In the UAE, national policies and accreditation standards increasingly prioritize research capacity within medical curricula. “We the UAE 2031” aims to invest in knowledge-based sectors and higher education, with student research as a critical component (5). The Ministry of Education’s Commission for Academic Accreditation now includes research as a key quality indicator for medical colleges, encouraging institutions to embed research into undergraduate programs (6). This aligns with the UAE’s goal to become a regional leader in health innovation, where future physicians are expected to deliver care and contribute to medical discovery and policy development.

The UAE has seven medical schools with programs differing in research emphasis, curriculum, and international affiliation. While most follow a similar structure of basic sciences followed by clinical rotations, research integration varies, with some requiring mandatory projects while others offering research as an elective. In some medical schools, a pre-clinical research module is mandated that includes theoretical training, practical skills and often culminates in a required curricular dissertation or publication-quality project. Medical schools’ curricula also adhere to the United Arab Emirates Competency Framework for Medical Education (UCFME), which includes Evidence Based Learning as a core competency. This approach integrates research skills into clinical responsibilities, known as Entrustable Professional Activities (EPA). Universities support this integration through dedicated facilities like the Center for Outcomes and Research in Education (CORE) apart from active Student Research Programs that provide mentorship, journal clubs, and resources to facilitate student involvement (7–9). It is also important to note that both MBBS and MD programs represent equivalent primary medical qualifications in the UAE, despite their nomenclatural differences (10, 11). MBBS and MD programs are similar in course length, lasting anywhere between 4 and 6 years. The student body is characterized by diverse nationalities, cultures, and academic backgrounds, making it an ideal context for examining factors influencing research engagement (12). Furthermore, the increasing demand for academic excellence in the UAE’s healthcare motivates students to develop strong research skills to meet institutional and career requirements (13). However, while a significant number of medical students engage in research, many still face substantial barriers, including a lack of mentorship, insufficient institutional support, and logistical challenges such as inadequate funding and limited access to research materials (14, 15).

Our study aims to explore attitudes, perceptions, and barriers to research among medical students in the UAE. By examining a large, diverse cohort, we seek to provide a comprehensive understanding of factors influencing research engagement and offer insights into strategies for improving research opportunities and support systems for future medical professionals.

## 2 Materials and methods

### 2.1 Study design

This study employed a cross-sectional observational exploratory design (16), involving the administration of a self-completed questionnaire distributed to medical students across seven medical colleges in the UAE.

### 2.2 Sampling and eligibility criteria

Inclusion criteria involved students enrolled in medical colleges across the UAE, studying in any year of Bachelor of Medicine, Bachelor of Surgery (MBBS), or Doctor of Medicine (MD) courses. The survey was shared across participating colleges from May through October 2024. We utilized purposive sampling to reach the target population efficiently. With a confidence interval of 95%, a margin of error of 5%, and a power of 80%, we set the appropriate minimum sample size of the study to 347 responses, which was calculated using the online Raosoft sample calculator (17).

### 2.3 Survey design and data collection

#### 2.3.1 Questionnaire development and validation

We distributed the self-administered online survey via Google Forms. The survey instrument was developed through a systematic process informed by extensive literature review of existing tools measuring research attitudes, barriers, and engagement among medical students and early-career healthcare professionals. Our review identified several validated instruments, including the Research Attitudes Questionnaire (18), the Barriers to Research scale used in Saudi medical schools (4, 19), and tools employed in regional studies examining research culture in medical education. While these instruments demonstrated strong psychometric properties, they were primarily developed in Western educational contexts or focused on specific aspects of research engagement without comprehensively addressing the unique institutional, cultural, and logistical challenges faced by medical students in the UAE (4, 19, 20).

We therefore adapted and expanded items from these established tools to create a context-specific instrument capturing the multidimensional nature of research engagement in the UAE medical education environment. The development process involved: (1) literature review of existing instruments; (2) item adaptation and generation to address UAE-specific barriers including funding constraints, institutional support structures, and diverse student populations; (3) expert panel review for content validity; and (4) pilot testing with medical students to assess clarity and comprehension.

#### 2.3.2 Content validity assessment

Content validity was rigorously evaluated using the Content Validity Index (CVI) methodology 21, 22). An expert panel independently evaluated each survey item across three dimensions: relevance, clarity, and appropriateness. Experts rated each item using a 4-point scale (1 = not relevant/clear/appropriate to 4 = highly relevant/clear/appropriate). Items rated 3 or 4 were considered valid.

Content validity was quantified using both item-level (I-CVI) and scale-level (S-CVI/Ave) indices. The questionnaire demonstrated excellent content validity across all dimensions. All 53 items achieved I-CVI scores of 1.00 for both relevance and appropriateness, indicating unanimous expert agreement. Scale-level content validity indices were: S-CVI/Ave for relevance = 1.00, clarity = 0.997, and appropriateness = 1.00. These values substantially exceed the recommended threshold of 0.90 (22), providing strong evidence for the instrument’s content validity.

#### 2.3.3 Reliability assessment

Internal consistency reliability was assessed using Cronbach’s alpha coefficient for the two primary subscales. The Barriers subscale (24 items) demonstrated excellent internal consistency (α = 0.959), while the Attitudes and Perceptions subscale (13 items) showed good internal consistency (α = 0.872). Both values exceed the recommended threshold of 0.70 for research instruments (23), indicating high reliability and supporting the questionnaire’s psychometric quality.

#### 2.3.4 Survey structure and scoring

The final validated questionnaire comprised 53 items distributed across four sections: (1) demographics (8 items including gender, nationality, age, medical college, year of study, academic stage, and cumulative GPA); (2) research experience and publishing status (5 items assessing prior project involvement, publications, and curriculum-based research training); (3) attitudes toward research (13 items); and (4) perceived barriers to research engagement (24 items). Attitude and barrier items utilized a 5-point Likert scale (1 = Strongly Disagree to 5 = Strongly Agree) with an additional “not applicable” option.

For descriptive analyses, responses are reported using the complete 5-point scale to preserve granularity and allow examination of response distributions. For inferential analyses, particularly multivariate regression models, we calculated composite attitude and barrier scores using a trichotomous scoring system: responses indicating agreement (Agree/Strongly Agree) were assigned + 1, Neutral responses = 0, and disagreement responses (Disagree/Strongly Disagree) = −1. This approach provides conceptually clearer interpretation of overall positive versus negative orientations and is consistent with established attitude measurement methodology, while the full 5-point distributions remain available in descriptive tables for detailed examination (24).

The complete questionnaire is provided as Supplementary material 1.

#### 2.3.5 Recruitment and sampling

We employed a purposive snowball sampling approach (25), recruiting medical student ambassadors from each of the seven UAE medical colleges to distribute the questionnaire through institutional communication channels, primarily WhatsApp groups, from May through October 2024.

The inclusion criteria for this study were students currently enrolled in any year of MBBS or MD programs at UAE medical colleges during the study period. The exclusion criteria comprised students enrolled in other health professions programs (non-medical students), visiting or exchange students not formally enrolled in UAE medical programs, and incomplete survey responses.

#### 2.3.6 Data quality control

To ensure data integrity and validity, two independent reviewers systematically screened the complete dataset for potential duplicates and data quality issues. The screening process examined timestamps, response patterns, and demographic consistency across all variables to identify any implausible or duplicate responses. The survey’s demographic questions, which included medical college affiliation, year of study, academic stage, and cumulative grade point average (CGPA), served as additional verification that all respondents were eligible medical students. This comprehensive quality assurance process resulted in a final verified sample of 612 participants representing all seven UAE medical colleges.

### 2.4 Statistical analysis

Data were analyzed using descriptive and inferential statistical methods. Descriptive statistics (means, standard deviations, and frequencies) summarized demographic characteristics, research participation, and perceived barriers. Inferential analyses included chi-square tests to assess associations between categorical variables, one-way ANOVA to compare group differences in continuous variables, and independent sample *t*-tests to compare research attitudes and experiences across key demographics. We conducted multivariate linear regression models to identify predictors of research attitudes, perceptions, motivation, participation, and barriers, reporting R-squared values and beta coefficients to evaluate model significance. Data analysis used SPSS version 29.0.2.0. *P* < 0.05 was considered statistically significant.

### 2.5 Ethical approval

The study was approved by the MBRU Institutional Review Board (Reference: MBRU IRB-2023–43) and Dubai Scientific Research Ethics Committee (DSREC) for Dubai Health Authority (Reference: DSREC-03/2024_16). All participants provided fully informed electronic consent before study commencement.

## 3 Results

### 3.1 Sample characteristics

The study included 612 medical students from seven universities across the UAE. The majority were male (68.8%, *n* = 421) with a mean age of 20.19 years (SD = 2.04; range: 16–30 years). Two-thirds (66.5%, *n* = 407) were in the pre-clinical stage. Overall, 68.3% (*n* = 418) had participated in research, but only 15.7% (*n* = 96) had published; systematic reviews (16.7%, *n* = 16) and case reports (13.5%, *n* = 13) predominated (Table 1). Students were predominantly from Eastern Mediterranean (53.4%, *n* = 327) and South-East Asia (33.0%, *n* = 202) regions, with the most common nationalities being India (31.7%), UAE (12.1%), and Syria (10.6%). Academically, 69.4% (*n* = 425) had a CGPA ≥ 3.1.

**TABLE 1:** Characteristics of the medical students (*n* = 612).

Characteristics	*n*	%	
**Gender**			
Male	421	68.8	
Female	191	31.2	
**Age**			
Age in years	20.19 ± 2.04 (16–30)		
≤ 17 years	34	5.6	
18–19 years	202	33.0	
20–21 years	241	39.4	
22–23 years	111	18.1	
24–25 years	11	1.8	
26–27 years	8	1.3	
28 + years	5	0.8	
**Nationality**			
**South-East Asia**	**202**	**33.0**	
Bangladesh	6	1.0	
India	194	31.7	
Sri Lanka	2	0.3	
**Africa**	**16**	**2.7**	
Algeria	2	0.3	
Angola	1	0.2	
Kenya	1	0.2	
Mauritius	1	0.2	
South Africa	1	0.2	
Sudan	10	1.6	
**Americas**	**37**	**6.0**	
Canada	18	2.9	
Dominica	3	0.5	
United States	14	2.3	
Venezuela	2	0.3	
**Europe**	**23**	**3.8**	
Armenia	1	0.2	
France	2	0.3	
Germany	3	0.5	
Hungary	1	0.2	
Italy	2	0.3	
Norway	1	0.2	
Turkey	1	0.2	
United Kingdom	12	2.0	
**Eastern Mediterranean**	**327**	**53.4**	
Afghanistan	3	0.5	
Egypt	31	5.1	
Iran	7	1.1	
Iraq	36	5.9	
Jordan	35	5.7	
Kuwait	15	2.5	
Lebanon	2	0.3	
Libya	1	0.2	
Morocco	1	0.2	
Pakistan	39	6.4	
Palestine	10	1.6	
Saudi Arabia	1	0.2	
Somalia	1	0.2	
Syria	65	10.6	
Tunisia	1	0.2	
United Arab Emirates	74	12.1	
Yemen	5	0.8	
**Western Pacific**	**7**	**1.2**	
Australia	4	0.7	
China	2	0.3	
Indonesia	1	0.2	
**Previous degree qualification**			
No	549	89.7	
Yes	63	10.3	
**Year of study**			
Year 1	158	25.8	
Year 2	102	16.7	
Year 3	118	19.3	
Year 4	111	18.1	
Year 5	102	16.7	
Year 6	21	3.4	
**Academic stage**			
Basic sciences (pre-clinical/pre-medical years)	407	66.5	
Clinical clerkship (clinical years)	205	33.5	
**CGPA**			
Not available	92	15.0	
2.00–2.5 (or 70–80%)	30	4.9	
2.6–3.00 (or 80–85%)	65	10.7	
3.1–3.5 (or 85–90%)	166	27.1	
3.6–4.00 (or 90–100%)	259	42.3	
**Previous participation in a research project**			
No	194	31.7	
Yes	418	68.3	
**Previous publication of a research article**			
No	516	84.3	
Yes	96	15.7	
**Type of research study published**			
Case control study	5	5.2	
Case report	13	13.5	
Case report, case control study	1	1.0	
Case report, case series	2	2.1	
Case report, case series, cohort study	1	1.0	
Case report, cohort study, meta-analysis	1	1.0	
Case report, systematic review	1	1.0	
Case report, systematic review, meta-analysis, other	1	1.0	
Case series	2	2.1	
Case series, cohort study	1	1.0	
Cohort study	6	6.3	
Cohort study, case control study	1	1.0	
Cohort study, case control study, systematic review, meta-analysis	1	1.0	
Cohort study, systematic review	2	2.1	
Meta-analysis	2	2.1	
Meta-analysis, other	1	1.0	
Other	31	32.3	
Systematic review	16	16.7	
Systematic review, meta-analysis	4	4.2	
Systematic review, meta-analysis, other	1	1.0	
Systematic review, other	3	3.1	
**Number of research publications during Medical training**			
0	519	84.8	
1	62	10.1	
2	14	2.3	
3	12	2.0	
4	2	0.3	
5	2	0.3	
10	1	0.2	
**Medical curriculum offering an educational course on research**			
No	106	17.3	
Yes	506	82.7	
**If yes, was the knowledge shared sufficient to participate in research projects?**			
No	100	19.8	
Yes	406	80.2	
**Intention to continue research after graduation**			
No	156	25.5	
Yes	456	74.5	
**Participation in research development activities (courses, seminars, workshops, etc.)**			
No	341	55.7	
Yes	271	44.3	
**Principal motivation for conducting research (multiple responses permitted)**			
**Motivation**	**n (selections)**	**% of students (*n* = 612)**	**% of selections (*n* = 1,233)**
To achieve academic excellence	356	58.2%	28.9%
For CV improvement purposes	210	34.3%	17.0%
Institutional requirement	173	28.3%	14.0%
Out of personal interest	226	22.7%	18.3%
Knowledge sharing	136	22.2%	11.0%
My supervisor encouraged me to	100	16.3%	8.1%
Peer pressure	32	5.2%	2.6%

Percentages are calculated with the respondent-level denominator (*n* = 612) unless otherwise specified and may not sum to 100% due to rounding. Please note that the “*Principal Motivation for Conducting Research”* subsection allowed multiple responses; for this subsection, we report both the percentage of students (*n* = 612) and the percentage of selections (total selections, *n* = 1,233). Row percentages in this subsection therefore exceed 100% by design. CV, curriculum Vitae; CGPA, Cumulative Grade Point Average. Region names in bold refer to World Health Organization (WHO) regions: Africa, the Americas, Eastern Mediterranean, Europe, South-East Asia and Western Pacific.

Primary research motivations were academic excellence (356 selections; 58.2% of students), followed by CV improvement (210; 34.3%) and institutional requirements (173; 28.3%). Multiple responses were permitted. While 82.7% (*n* = 506) had access to research courses as part of their medical training, 19.8% (*n* = 100) found the knowledge insufficient. Future engagement in research was mixed; 25.5% (*n* = 156) reported no intention to continue research after graduation. Participation in research development activities was moderate (44.3%, *n* = 271).

### 3.2 Attitudes and perceptions toward research

Students expressed positive overall attitudes. 91.0% (*n* = 557) agreed research should be integrated into the medical curriculum (Table 2). A significant proportion recognized research as crucial for career evaluations (88.4%, *n* = 541) and CV building (91.0%, *n* = 557). Additionally, 94.1% (*n* = 576) believed research benefits regional scientific advancement, while 63.1% (*n* = 386) agreed research influences clinical practice and guidelines.

**TABLE 2:** Student attitudes and perceptions toward research (*n* = 612).

Statement	Strongly agree	Agree	Neutral	Disagree	Strongly disagree	Not applicable
Research should be taught to all medical students as part of their curriculum	386 (63.1)	171 (27.9)	42 (6.9)	9 (1.5)	4 (0.7)	–
Research is an important factor in future career evaluations and CV	357 (58.3)	184 (30.1)	53 (8.7)	11 (1.8)	6 (1.0)	1 (0.2)
Research is an important factor in building a holistic CV	376 (61.4)	181 (29.6)	38 (6.2)	11 (1.8)	5 (0.8)	1 (0.2)
The skills I have acquired in research will be helpful to me in my future career	303 (49.5)	217 (35.5)	68 (11.1)	13 (2.1)	9 (1.5)	2 (0.3)
Conducting research is beneficial to the region’s scientific advancement	411 (67.2)	165 (27.0)	30 (4.9)	2 (0.3)	2 (0.3)	2 (0.3)
Research can influence clinical practice and guidelines	386 (63.1)	189 (30.9)	29 (4.7)	3 (0.5)	2 (0.3)	3 (0.5)
Research can be interesting	318 (52.0)	194 (31.7)	80 (13.1)	9 (1.5)	8 (1.3)	3 (0.5)
I enjoy conducting research	230 (37.6)	197 (32.2)	129 (21.1)	22 (3.6)	28 (4.6)	6 (1.0)
I feel validated when publishing research	294 (48.0)	187 (30.6)	100 (16.3)	7 (1.1)	6 (1.0)	18 (2.9)
Engaging in research poses challenges	262 (42.8)	253 (41.3)	76 (12.4)	13 (2.1)	5 (0.8)	3 (0.5)
I struggle to grasp research concepts	104 (17.0)	146 (23.9)	187 (30.6)	84 (13.7)	74 (12.1)	17 (2.8)
I lack confidence in conducting statistical analysis for research	102 (16.7)	148 (24.2)	168 (27.5)	92 (15.0)	81 (13.2)	21 (3.4)
I feel pressured to publish research for the sake of career progression	171 (27.9)	176 (28.8)	128 (20.9)	75 (12.3)	48 (7.8)	14 (2.3)
Research is irrelevant to my current stage of medical education	92 (15.0)	92 (15.0)	121 (19.8)	100 (16.3)	136 (22.2)	71 (11.6)

Please note that percentages may not sum to 100% due to rounding. CV, Curriculum Vitae.

Research interest varied, with 83.7% (*n* = 512) finding it interesting and 70.0% (*n* = 427) reporting enjoyment in conducting research. Additionally, 78.6% (*n* = 481) felt validated by publishing. However, 42.8% (*n* = 262) strongly agreed and 41.3% (*n* = 253) agreed that research is challenging.

Confidence in research skills was mixed; 17.0% (*n* = 104) strongly agreed and 23.9% (*n* = 146) agreed they struggled with research concepts, while 16.7% (*n* = 102) strongly agreed and 24.2% (*n* = 148) agreed they lacked confidence in statistical analysis. Pressure to publish was prominent; 16.7% (*n* = 102) strongly agreed and 28.8% (*n* = 176) agreed that career progression requires publications. Regarding relevance, 22.2% (*n* = 136) strongly disagreed research was irrelevant to their education stage, while 19.8% (*n* = 121) remained neutral on its applicability.

### 3.3 Perceived research barriers

Institutional and logistical challenges were notable (Table 3). Lack of funding was the most commonly reported barrier, with 20.6% (*n* = 126) strongly agreeing and 25.8% (*n* = 158) agreeing that it hindered research engagement. Lack of protected research time was a concern for nearly half of the students (46.9%, *n* = 287).

**TABLE 3:** Potential research barriers and challenges amongst students (*n* = 612).

Barrier	Strongly agree	Agree	Neutral	Disagree	Strongly disagree	Not applicable
Lack of supportive environment in the institution to conduct research	71 (11.6)	110 (18.0)	146 (23.9)	197 (32.2)	73 (11.9)	15 (2.5)
Difficulty in obtaining institutional review board (IRB) approval	72 (11.8)	135 (22.1)	200 (32.7)	108 (17.6)	56 (9.2)	41 (6.7)
Lack of mentorship	93 (15.2)	125 (20.4)	132 (21.6)	172 (28.1)	66 (10.8)	24 (3.9)
Lack of encouragement of researchers to conduct research	80 (13.1)	131 (21.4)	131 (21.4)	180 (29.4)	67 (10.9)	23 (3.8)
Lack of physical/virtual space to conduct research	67 (10.9)	144 (23.5)	139 (22.7)	173 (28.3)	67 (10.9)	22 (3.6)
Lack of access to research articles/digital resources	69 (11.3)	75 (12.3)	140 (22.9)	204 (33.3)	96 (15.7)	28 (4.6)
Lack of access to laboratory equipment for performing a research project	85 (13.9)	132 (21.6)	147 (24.0)	147 (24.0)	67 (10.9)	34 (5.6)
Lack of protected research time	112 (18.3)	175 (28.6)	142 (23.2)	104 (17.0)	54 (8.8)	25 (4.1)
Lack of funding	126 (20.6)	158 (25.8)	160 (26.1)	93 (15.2)	39 (6.4)	36 (5.9)
Lack of collaboration between research centers	105 (17.2)	167 (27.3)	152 (24.8)	108 (17.6)	41 (6.7)	39 (6.4)
Lack of equal opportunities between the students	118 (19.3)	138 (22.5)	151 (24.7)	118 (19.3)	68 (11.1)	19 (3.1)
Lack of motivation or interest to conduct research	85 (13.9)	156 (25.5)	136 (22.2)	140 (22.9)	74 (12.1)	21 (3.4)
Lack of financial incentive to conduct research projects	97 (15.8)	152 (24.8)	167 (27.3)	108 (17.6)	61 (10.0)	27 (4.4)
Lack of confidence in starting a research project	88 (14.4)	183 (29.9)	144 (23.5)	111 (18.1)	64 (10.5)	22 (3.6)
Lack of familiarity with research studies	103 (16.8)	175 (28.6)	147 (24.0)	106 (17.3)	58 (9.5)	23 (3.8)
Lack of familiarity with conducting statistical analysis	117 (19.1)	175 (28.6)	129 (21.1)	108 (17.6)	60 (9.8)	23 (3.8)
Lack of skills necessary for the process of submitting research articles	113 (18.5)	174 (28.4)	136 (22.2)	106 (17.3)	63 (10.3)	20 (3.3)
Lack of familiarity with research proposal writing	94 (15.4)	167 (27.3)	147 (24.0)	117 (19.1)	64 (10.5)	23 (3.8)
Lack of innovative research ideas	103 (16.8)	148 (24.2)	140 (22.9)	133 (21.7)	65 (10.6)	23 (3.8)
Lack of academic recognition to pursue research	82 (13.4)	136 (22.2)	157 (25.7)	147 (24.0)	63 (10.3)	27 (4.4)
Cultural barriers between collaborators	61 (10.0)	80 (13.1)	177 (28.9)	150 (24.5)	104 (17.0)	40 (6.5)
Time zone or location barriers between collaborators	64 (10.5)	79 (12.9)	185 (30.2)	132 (21.6)	102 (16.7)	50 (8.2)

Please note that percentages may not sum to 100% due to rounding.

Mentorship and institutional support issues were also noticeable. 15.2% (*n* = 93) strongly agreed and 20.4% (*n* = 125) agreed lack of mentorship was a barrier. Similarly, lack of encouragement (13.1%, *n* = 80 strongly agreed; 21.4%, *n* = 131 agreed) and lack of supportive research environment (11.6%, *n* = 71 strongly agreed; 18.0%, *n* = 110 agreed) were frequently cited.

Access-related barriers showed varying patterns. Obtaining Institutional Review Board (IRB) approval was difficult for 33.8% (*n* = 207), and 35.5% (*n* = 217) felt limited by a lack of laboratory equipment. However, access to research articles/digital resources was less problematic; only 23.6% (*n* = 144) reported limitations, while 33.3% (*n* = 204) disagreed that this was a barrier.

Skills and knowledge gaps were evident. 44.3% (*n* = 271) agreed they lacked confidence starting research projects, and 45.4% (*n* = 278) struggled with research study familiarity. Lack of statistical analysis skills was a major concern; 28.6% (*n* = 175) agreed, while 19.1% (*n* = 117) strongly agreed.

Motivation-related challenges that hindered engagement in research included lack of motivation (25.5%, *n* = 156 agreed; 13.9%, *n* = 85 strongly agreed) and lack of financial incentives (24.8%, *n* = 152 agreed; 15.8%, *n* = 97 strongly agreed). Collaboration barriers included lack of equal opportunities (19.3%, *n* = 118 strongly agreed; 22.5%, *n* = 138 agreed) and lack of collaboration between research centers (17.2%, *n* = 105 strongly agreed; 27.3%, *n* = 167 agreed). Cultural barriers between collaborators (23.0%, *n* = 141) and time zone differences (23.4%, *n* = 143) were also noted.

### 3.4 Inferential statistics

Chi-square tests examined associations between demographics and research factors. Age was significantly associated with multiple factors. Younger students engaged in research for CV-building, whereas older students focused on knowledge contribution (*p* < 0.001). Students ≤ 21 years perceived curriculum knowledge as sufficient (*p* = 0.002) and were inclined to pursue research after graduation (*p* = 0.002), but expressed highest concerns regarding lack of mentorship (*p* = 0.009) and absence of academic recognition (*p* = 0.021). Older students (≥ 24 years) reported greater difficulties balancing research and perceived funding lack as a barrier (*p* < 0.001). Participation in research development activities was lowest among the youngest and oldest students, peaking at ages 20–23 (*p* = 0.002). A trend was observed where older students were more likely to engage in research activities and subsequently publish their findings (*p* = 0.001).

Gender differences were significant. Males were more likely to view financial constraints as limiting (*p* = 0.007) and felt more pressure to publish for career advancement (*p* = 0.021). Females were more likely to report perceived lack of mentorship (*p* = 0.044), lack of encouragement (*p* = 0.07), and demonstrated lower research motivation (*p* = 0.028). No significant associations existed between gender and perceived knowledge sufficiency (*p* = 0.472), intention to continue research (*p* = 0.472), or participation in development activities (*p* = 0.651).

Year of study showed senior students (years 4–6) had significantly higher publishing likelihood (*p* < 0.001) but encountered greater challenges balancing research and academics (*p* < 0.001) and securing funding (*p* = 0.001). Students in earlier years (years 1–3) are more likely to express intent to pursue research (*p* = 0.003). A significant relationship existed between year and participation in development activities (*p* = 0.002), indicating greater engagement among pre-clinical students. Clinical students reported slightly lower access to structured research training (*p* = 0.005) and were more likely to perceive deficiencies in research education (*p* = 0.054). Clinical students were more likely to publish systematic reviews and case reports (*p* < 0.001).

Higher CGPA students (3.6–4.0) were more likely to be engaged in research (*p* < 0.001) but expressed concerns about networking opportunities (*p* = 0.045). Previous degree qualification was not significantly associated with perceived knowledge sufficiency (*p* = 0.725) or intention to continue research (*p* = 0.725).

Independent sample *t*-tests showed significant differences (*p* < 0.001) across age, gender, nationality, previous degree, year of study, academic stage, CGPA, previous research participation, previous publication, number of publications, research course in curriculum, perceived knowledge sufficiency, intention to continue research, and participation in development activities. Attitudes and Perception Scores (*p* < 0.001) and Research Barriers Scores (*p* = 0.035) showed significant differences across these variables.

One-way ANOVA assessed demographic and academic variables’ influence on scores. For Attitudes and Perception scores, significant associations existed with age (*p* = 0.012), type of research study published (p = 0.013), and number of publications (*p* = 0.043). For Research Barriers scores, significant associations existed with age (*p* < 0.001), nationality (*p* = 0.037), year of study (*p* < 0.001), academic stage (*p* = 0.039), CGPA (*p* = 0.043), and previous project participation (*p* < 0.001) (Table 4).

**TABLE 4:** Results of the *t*-test and ANOVA analyses assessing research participation, attitudes, and barriers among medical students.

Variable						Mean difference ± SD		*p*-value		
*t*-test										
Age (years)						20.181 ± 1.019		<0.001*		
Gender						0.688 ± 0.464		<0.001*		
Nationality						3.446 ± 1.850		<0.001*		
Previous degree qualification						0.103 ± 0.304		<0.001*		
Year of study						2.935 ± 1.535		<0.001*		
Academic stage						1.335 ± 0.472		<0.001*		
CGPA						3.889 ± 2.226		<0.001*		
Previous participation in research						0.683 ± 0.466		<0.001*		
Previous publication						0.157 ± 0.364		<0.001*		
Number of research publications						0.252 ± 0.777		<0.001*		
Research course in curriculum						0.827 ± 0.379		<0.001*		
Perceived sufficiency of research knowledge						1.010 ± 0.581		<0.001*		
Intention to continue research after graduation						1.010 ± 0.581		<0.001*		
Participation in research development activities						0.443 ± 0.497		<0.001*		
Attitudes and perception score						0.63 ± 0.260		<0.001*		
Research barriers score						0.04 ± 0.575		0.035*		
	**Attitudes and perception score**					**Research barriers score**					
	**Sum of squares**	**df**	**Mean square**	***F***	***p*-value**	**Sum of squares**	**df**	**Mean square**	***F***	***p*-value**
ANOVA										
Age (years)	37.989	20	1.899	1.88	0.012*	220.902	83	2.661	3.4	<0.001*
Gender	3.622	20	0.181	0.84	0.668	15.181	83	0.183	0.83	0.851
Nationality	58.357	20	2.918	0.85	0.654	360.674	83	4.345	1.33	0.037*
Year of study	60.743	20	3.037	1.3	0.17	483.958	83	5.831	3.22	<0.001*
Academic stage	5.231	20	0.262	1.18	0.266	23.436	83	0.282	1.32	0.039*
CGPA	130.054	20	6.503	1.33	0.155	517.442	83	6.234	1.31	0.043*
Previous participation in a research project	2.528	20	0.126	0.58	0.93	27.604	83	0.333	1.67	<0.001*
Previous publication of a research article	3.25	20	0.163	1.24	0.218	9.175	83	0.111	0.81	0.878
Type of research study published	518.607	20	25.93	1.86	0.013*	1013.92	83	12.216	0.83	0.848
Number of research publications during medical training	19.171	20	0.959	1.62	0.043*	50.84	83	0.613	1.02	0.447
Attitudes and perception score	–	–	–	–	–	8.733	83	0.105	1.71	<0.001*
Research barriers score	24.353	20	1.218	4.05	<0.001*	–	–	–	–	–

The *t*-test results present the mean difference ± standard deviation (SD) for key demographic and research-related variables. The ANOVA results compare differences across multiple groups for Attitudes and Perception Score and Research Barriers Score, reporting sum of squares, degrees of freedom (df), mean square, F-statistic, and *p*-values (*n* = 612). Statistically significant findings (*p* < 0.05) are indicated with an asterisk (*). SD: standard deviation; df: Degrees of Freedom; F: F-statistic. CV, Curriculum Vitae; CGPA, Cumulative Grade Point Average.

Multivariate linear regression determined factors influencing research attitudes, participation, and barriers (Table 5). Age (*p* = 0.024), time zone barriers (*p* = 0.036), and research barriers score (*p* < 0.001) significantly influenced attitudes. Nationality (*p* < 0.001), medical college (*p* = 0.001), year of study (*p* = 0.042), CGPA (*p* = 0.019), previous publication (*p* = 0.004), and participation in research activities (*p* = 0.001) were strong predictors of previous participation. Academic stage (*p* = 0.036), number of publications (*p* = 0.036), and educational course availability (*p* = 0.010) significantly influenced motivation. Research barriers were significantly associated with IRB approval difficulty (*p* < 0.001), attitudes/perceptions (*p* < 0.001), and lack of access to research resources (*p* = 0.035).

**TABLE 5:** Predictors of medical students’ attitudes, perceptions, previous participation in research projects, principal motivation for conducting research, and research barrier scores.

Variable	R-squared	Regression coefficient (Beta)	*p*-value
**Attitudes and perceptions toward research**			
Age (years)	0.188	-0.374	0.024*
Difficulty in obtaining Institutional Review Board (IRB) approval		-0.094	0.074
Time zone or location barriers between collaborators		0.125	0.036*
Research is irrelevant to my current stage of medical education		0.041	0.396
Lack of supportive environment in the institution to conduct research		-0.065	0.208
Research barriers score		0.286	<0.001*
**Previous participation in a research project**			
Nationality	0.349	0.279	<0.001*
Medical college		0.145	0.001*
Year of study		0.081	0.042*
CGPA		0.089	0.019*
Previous publication of a research article		0.221	0.004*
Participation in research development activities		0.134	0.001*
Intention to continue research after graduation		0.117	0.046*
Medical curriculum offering an educational course on research		0.174	0.004*
**Principal motivation for conducting research**			
Academic stage	0.160	-6.678	0.036*
Number of research publications during medical training		3.525	0.036*
Medical curriculum offering an educational course on research		9.564	0.010*
Intention to continue research after graduation		6.144	0.009*
I feel validated when publishing research		1.452	0.015*
Engaging in research poses challenges		-1.021	0.050*
Lack of access to research articles/digital resources		-1.491	0.035*
Lack of academic recognition to pursue research		2.061	0.008*
**Research barriers**			
Participation in research development activities	0.148	0.14	0.003*
Conducting research is beneficial to the region’s scientific advancement		-0.089	0.127
Research is irrelevant to my current stage of medical education		-0.114	0.016*
Difficulty in obtaining institutional review board (IRB) approval		0.232	<0.001*
Lack of mentorship		-0.107	0.066
Attitudes and perceptions score		0.273	<0.001*

Please note that statistically significant findings (*p* < 0.05) are indicated with an asterisk (*).

## 4 Discussion

### 4.1 Key findings

This nationwide multi-center study provides a thorough insight into the attitudes, perceptions, and barriers related to research among medical students in the UAE. Drawing on responses from 612 students spanning all years of study and multiple universities, it presents a comprehensive overview of the current landscape.

The study revealed positive attitudes from many students; however, significant barriers existed, including a lack of funding, mentorship, and research resources. Although 68.3% (*n* = 418) participated in research, only 15.7% (*n* = 96) published, with systematic reviews and case reports most common. Factors enabling engagement included academic excellence and CV enhancement, yet students faced institutional and logistical challenges. Inferential analyses revealed that age, gender, CGPA, and year of study significantly influenced research attitudes, participation, and perceived barriers. Regression models identified age, research barriers, collaboration-related constraints, time-zone barriers, and access to research training as key predictors of attitudes and involvement.

### 4.2 Regional and international context

Our findings reveal a cohort of medical students who are highly motivated to engage in research yet constrained by uneven skills, limited confidence, and structural barriers such as insufficient mentorship and funding. This pattern of high enthusiasm coupled with practical limitations aligns with findings from regional multi-center and single-country studies, despite contextual differences in curricula and support models (26–28).

Similar trends have been observed in Bangladesh, South Africa, and Saudi Arabia, where students likewise perceive research as integral to medical careers and professional growth (19, 29–31). For instance, Nel et al. surveyed 1195 University of Cape Town medical students, finding 61% held positive attitudes toward research, substantially lower than the proportion observed in our study (> 90%) (29). Similarly, Noorelahi et al. studied 223 students at Taibah University, Saudi Arabia, with over 70% recognizing research as crucial for clinical practice and its positive implications for patient outcomes, similar to our findings, although their study included a balanced proportion of male and female students, whereas our study had a larger proportion of male respondents (19).

### 4.3 Motivation and barriers

Positive attitudes stemmed from recognizing research as crucial for career evaluations and CV building, and research’s influence on clinical practice and guidelines. However, Queen’s University students perceived research differently—as a way to develop critical appraisal, information literacy, and critical thinking skills, and as an opportunity to identify future training pathways and form contacts (32).

Our multivariate regression revealed that academic stage, number of publications, and educational course availability significantly influenced motivation. AlSubai et al.’s Qatar cross-sectional study identified age > 22 years, male gender, and clinical year as strongly associated with extensive research participation (33). A larger multicenter study of 2989 students from Egypt, Algeria, Sudan, Jordan, Syria, and Palestine reported poor research knowledge but high positive attitudes (15). A key difference was that > 80% of our population reported sufficient knowledge to participate (among students whose curriculum included a research course), while Assar et al. reported overall poor knowledge regarding research (91.6%). Both studies found that over two-thirds of participants were involved in research (15).

Many students recognized research’s significance for career progression and scientific advancement; however, barriers included a lack of funding, insufficient mentorship, a lack of protected research time, a lack of confidence in research skills, and struggles with concepts and statistical analysis. These findings mirror El Achi et al.’s 2019 cross-sectional study of 523 American University of Beirut students (34). Interestingly, faculty at two major Indian institutions, All India Institute of Medical Sciences (AIIMS) and University College of Medical Sciences, also reported barriers, including a lack of financial support, statistical courses, and workshops (35).

A Jordan single-site cross-sectional survey revealed similarly positive attitudes regarding research integration into curricula. A substantial majority disagreed that research was time-wasting, and 88.5% believed research experience would benefit future clinical practice (20). Identified barriers aligned with ours, emphasizing insufficient training, limited opportunities, and inadequate faculty support. Comparable results in the broader MENA region included Orebi et al.’s Tanta University, Egypt study reporting strong student support for embedding structured research training within medical curricula, underscoring consistent regional recognition of research’s value and need to address barriers (36).

### 4.4 Implications

Early, structured training plus formal mentorship and protected research time are likely to yield the greatest gains. Medical students perceive research development activities, such as courses, workshops, and conferences, as valuable for academic and professional growth. These opportunities provide practical skills in research design, data analytics, and scientific writing, complementing coursework. Such opportunities help students develop autonomy, competence, and positive research perception in clinical contexts, encouraging research during training (37). This study is even more relevant to the UAE, where the transition to Competency Based Medical Education, demands that research competencies are attained not just theoretically but practically. Moreover, our results reveal critical stage specific needs showing that younger pre-clinical students are primarily CV driven and require basic structured mentorship while older students are more concerned with access to funding and resources to complete advanced projects. These observed differences in motivations and barriers based on age and gender (e.g., males citing funding, females citing mentorship) necessitate a differentiated, formalized mentorship model and the creation of dedicated undergraduate research grant programs to ensure equitable and effective research training that is explicitly linked to the national healthcare vision and the UCFME (8, 9).

### 4.5 Strengths and limitations

Key strengths include this study’s status as a nationwide, multi-site survey among UAE medical students, encompassing respondents from diverse universities, nationalities, and academic backgrounds. Large sample size provides statistical robustness, enhancing validity and generalizability. Given the existing scarcity of comprehensive data on medical students’ research attitudes, perceptions, and barriers in the MENA region, our study fills a critical knowledge gap, laying the groundwork for targeted institutional interventions and educational strategies. Importantly, this study focuses exclusively on medical students, whereas Jarab et al. (26) examined mixed health professions students. This enables a comprehensive analysis of the identification of barriers specific to medical curricula and their training demands. Our multivariate regression analyses identify independent predictors of research attitudes and barriers, including age, institutional factors, prior experience, and academic performance. These findings provide evidence for targeted medical education interventions rather than generalized recommendations across health professions.

When it comes to limitations, first, our methodology relied on a convenience sampling approach through voluntary, non-compulsory participation, which introduces a potential self-selection bias. Students who are already more engaged or interested in research may have been more inclined to complete the survey, potentially inflating the overall reported positive attitude and participation rates. We attempted to mitigate this by engaging multiple medical colleges across the UAE to ensure a diverse, nationally representative sample across various years of study and curriculum structures. Next, the use of a self-administered electronic questionnaire carries the risk of recall bias and social desirability bias, students may have inaccurately reported past activities or provided answers they perceived as socially or academically favorable. However, the anonymous nature of the data collection was implemented specifically to encourage candor and reduce this bias, particularly when responding to sensitive questions about specific skill deficiencies, such as statistics or personal barriers, such as a lack of mentorship. Finally, being cross-sectional, this study captures attitudes and perceptions at a single point in time and cannot establish causality through the research training journey. Future longitudinal studies would be necessary to evaluate the true impact of current medical curricula reforms on research participation.

## 5 Conclusion

UAE medical students value research but face consistent structural and skills-related barriers. Early, structured training plus formal mentorship and protected research time are likely to yield the greatest gains. Institutions should embed these elements longitudinally within undergraduate programs. Addressing inadequate mentorship, limited funding, and insufficient institutional support could help students become more actively engaged and productive in research. Medical schools should adopt more structured approaches to incorporating research into curricula, helping students build the skills and confidence necessary to meaningfully participate in research activities during training and future careers.

## Data Availability

The original contributions presented in this study are included in this article/Supplementary material. Further inquiries can be directed to the corresponding author.
