# Supplementary material for: Perceptions, attitudes, and barriers toward research among medical students in the United Arab Emirates: a national cross-sectional study
Source: Front Med (Lausanne). 2025 Dec 15;12:1729448. doi: 10.3389/fmed.2025.1729448 (PMC12745252; doi:10.3389/fmed.2025.1729448)
Supplement: Supplementary file 1 [file Data_Sheet_1.docx]

**Supplementary Material 1: Study Questionnaire**

**Title:** Perceptions, Attitudes, and Barriers Towards Research Among Medical Students in the United Arab Emirates: A National Cross-Sectional Study

**Corresponding Author:**Syed Ali Bokhari
[sab_191@live.com](mailto:sab_191@live.com)

Institutional Address: Al Amal Psychiatric Hospital, Emirates Health Services, Dubai, United Arab Emirates

*ORCID:* 0009-0009-1190-7613

| **Section 1: Demographic Characteristics** | |
| --- | --- |
| **1** | Your gender  (options: Male, Female) |
| **2** | Nationality  (Dropdown menu of countries) |
| **3** | Please indicate your age: ________ (years) |
| **4** | Do you have a previous degree qualification?  (options: Yes, No) |
| **5** | Please choose your medical college: |
| **6** | Year of study  (options: Year 1, Year 2, Year 3, Year 4, Year 5, Year 6). |
| **7** | Please indicate the academic stage you are currently in  (options: Basic Sciences / Pre-clinical / Pre-medical years; Clinical Clerkship / Clinical years). |
| **8** | Your cGPA  (options: < 2.00 or <70%; 2.00–2.5 or 70–80%; 2.6–3.00 or 80–85%; 3.1–3.5 or 85–90%; 3.6–4.00 or 90–100%). |
| **Section 2: Exploring Publishing Status of Medical Students** | |
| **9** | Have you ever participated in a research project before? (options: Yes, No) |
| **10** | Have you previously published a research article? (options: Yes, No) |
| **11** | If yes, how many research publications have you published during your medical training? ________ |
| **12** | What was your principal motivation to conduct research?  (*Multiple answers allowed*:  To achieve academic excellence; Out of personal interest; For CV improvement purposes; Institutional requirement; Peer pressure; Knowledge sharing; Supervisor encouraged me; Other – please state.) |
| **13** | Did your medical curriculum offer an educational course on research?  (options: Yes, No) |
| **14** | If yes, did you find the knowledge shared sufficient to participate in research projects?  (options: Yes, No) |
| **15** | Do you plan to continue participating in research projects after graduation?  (options: Yes, No) |
| **16** | Have you previously attended or participated in research development activities (courses, seminars, workshops, etc.)?  (options: Yes, No) |
| **Section 3: Student Attitude and Perception Towards Research** (Likert items) | |
| **17** | Research should be taught to all medical students as part of their curriculum. |
| **18** | Research is an important factor in future career evaluations and CV. |
| **19** | The skills I have acquired in research will be helpful to me in my future career. |
| **20** | Conducting research is beneficial to the region’s scientific advancement. |
| **21** | Research can influence clinical practice and guidelines. |
| **22** | Research can be interesting. |
| **23** | I enjoy conducting research. |
| **24** | I feel validated when publishing research. |
| **25** | The process of conducting research is difficult. |
| **26** | I find it difficult to understand the concepts of research. |
| **27** | I feel insecure about the statistical analysis of research data. |
| **28** | I feel pressured to publish research for the sake of career progression. |
| **29** | Research is irrelevant to my current stage of medical education. |
| **Section 4: Potential Barriers and Challenges Amongst Students** (Likert items) | |
| ***Institution-related barriers*** | |
| **30** | Lack of supportive environment in the institution to conduct research. |
| **31** | Difficulty in obtaining Institutional Review Board (IRB) approval. |
| **32** | Lack of mentorship. |
| **33** | Lack of encouragement of researchers to conduct research. |
| **34** | Lack of support given to researchers. |
| **35** | Lack of physical/virtual space to conduct research. |
| **36** | Lack of access to research articles / digital resources. |
| **37** | Lack of access to laboratory equipment for performing a research project. |
| **38** | Lack of protected research time. |
| **39** | Lack of funding. |
| **40** | Lack of collaboration between research centers. |
| **41** | Lack of equal opportunities between the students. |
| ***Individual-related barriers*** | |
| **42** | Lack of motivation or interest to conduct research. |
| **43** | Lack of financial incentive to conduct research projects. |
| **44** | Lack of confidence in starting a research project. |
| **45** | Lack of familiarity with research studies. |
| **46** | Lack of familiarity with conducting statistical analysis. |
| **47** | Lack of research writing skills. |
| **48** | Lack of skills necessary for the process of submitting research articles. |
| **49** | Lack of familiarity with research proposal writing. |
| **50** | Lack of innovative research ideas. |
| **51** | Lack of academic recognition to pursue research. |
| **52** | Cultural barriers between collaborators. |
| **53** | Time zone / location barriers between collaborators. |
